# Doing Good Again? A Multilevel Institutional Perspective on Corporate Environmental Responsibility and Philanthropic Strategy

**DOI:** 10.3390/ijerph14101283

**Published:** 2017-10-24

**Authors:** Wei Liu, Qiao Wei, Song-Qin Huang, Sang-Bing Tsai

**Affiliations:** 1 Zhongshan Institute, University of Electronic Science and Technology of China, Zhongshan 528402, China; 2 Discipline of International Business, The University of Sydney, Sydney 2006, Australia; dqytliu@gmail.com; 3 Faculty of Education, Monash University, Melbourne 3800, Australia; 4 School of Economics and Management, Southeast University, Nanjing 211189, China; sqhuang0704@163.com

**Keywords:** corporate philanthropy, government intervention, government corruption, resource dependence, institutional environment, multilevel analysis

## Abstract

This study investigates the relationship between corporate environmental responsibility and corporate philanthropy. Using a sample of Chinese listed firms from 2008 to 2013, this paper examines the role of corporate environmental responsibility in corporate philanthropy and the moderating influence of the institutional environment using multilevel analysis. The results show that corporate eco-friendly events are positively associated with corporate philanthropic strategy to a significant degree. Provincial-level government intervention positively moderate the positive relationship between eco-friendly events and corporate philanthropy and government corruption is negatively moderate the relationship. All these results are robust according to robustness checks. These findings provide a new perspective on corporate philanthropic strategy as a means to obtain critical resources from the government in order to compensate for the loss made on environmental responsibility. Moreover, the institutional environment is proved here to play an important role in corporate philanthropic strategy.

## 1. Introduction

Corporate philanthropy has become an increasingly important subject of academic literature in recent years. Regarding the output of corporate philanthropy, scholars originally focused on corporate financial sustainability (e.g., stakeholder management or, financial performance), and gradually shifted their focus to environmental issues as philanthropic studies developed and environmental awareness increased in wider society [1,2,3,4]. Study of corporate philanthropy and environmental studies have developed in interaction with one another and the notion of “corporate environmental responsibility” is becoming an indispensable part of corporate research. Corporate environmental responsibility focuses on firm-specific activities taken by firms to limit the harmful impact they have on the environment and to strengthen sustainable development beyond compliance with legal requirements [5,6,7].

A growing body of literature has studied several aspects of the outcomes of corporate environmental responsibility. Based on the significant relationship between corporate philanthropy and corporate financial performance, corporate environmental responsibility was expected to be related to financial performance [8]. Ambec and Lanoie, Dollinger, King and Lenox examined the relationship between corporate environmental responsibility and financial performance from different perspectives and at different points in time [9,10,11]. Moreover, corporate environmental responsibility has been proven to play an important role in corporate sustainable development from the perspectives of stakeholder management, organizational size and investor willingness [3,4,12,13]. However, compared with the large number of studies on the outcome of corporate environmental responsibility, the relationship between corporate environmental responsibility and corporate philanthropy has been paid relatively little attention. Du focused on corporate environmental misconduct and found a positive relationship between corporate environmental misconduct and corporate philanthropy [14]. Because corporate philanthropy can alleviate negative perceptions of a firm, environmental wrongdoers often use this as a corporate philanthropic strategy to overshadow and draw attention away from their wrongdoing. To fill the existing research gap, it should be made clear how corporate philanthropic strategy is affected for firms doing good work for the environment. Resource dependence theory has well noted the role that dependence on resources has on corporate survival and development [15,16,17]. As a focus on environmental responsibility causes firms to consume excess resources, they select specific strategies in order to obtain the resources necessary for their development. In the institutional environment of China, critical resources are controlled by the government (both central and local), which also controls how these are dispensed and provided [18,19,20]. Thus, firms need to meet the government’s expectations and gain political capital to exchange for critical resources. This may affect corporate philanthropic strategies. 

Based on these reviews and the research gap above, the brief research questions in this study are clear: How corporate behavior on eco-friendly events affects corporate philanthropic strategy and how this relationship to be confirmed is affected by the institutional environment? According to these research questions, using a panel dataset of 2516 Chinese listed firms, we aim to address this issue by examining corporate environmental responsibility and the way such environmental responsibility affects the extent to which firms engage in corporate philanthropic strategy. Previous literature has suggested that firms with a higher-level intensity of eco-harmful events are more motivated to participate in corporate philanthropy in order to alleviate the negative influence these eco-harmful events have on the way they are perceived [14,21]. We go step further and argue that firms with a high-level intensity of eco-friendly events have a positive correlation to corporate philanthropy. Furthermore, this study offers a more precise description of the relationship between corporate eco-friendly events and corporate philanthropy by considering the moderating roles of government intervention and government corruption. Thus, this study introduces new evidence for why firms with higher level of environmental responsibility engage in corporate philanthropy and why the institutional environment significantly impacts such corporate strategies.

This study contributes several perspectives to the existing literature. Firstly, to the best of our knowledge and the literature in hand, this study is one of the few to study the relationship between corporate environmental responsibility and corporate philanthropy, or to put it more specifically, the relationship between corporate eco-friendly events and corporate philanthropy, from an empirical perspective. Thus, this study can provide a new angle of view in the analysis of corporate philanthropy, and broaden the study scope of the relationship between corporate environmental responsibility and corporate philanthropy. Secondly, we found that the Chinese institutional environment, including government intervention and government corruption, plays an important role in the study of corporate philanthropy. Finally, regarding our research methods, our study brings the hierarchical structures of firms and governmental institutions into corporate philanthropic studies to better reveal institutional effects on corporate responsibility.

The remainder of this study is organized as follows: the next section provides a development of our hypotheses and provides the details of our theoretical arguments. The third section describes our data and the methodology of multilevel analysis. The empirical results are then presented, and our discussions and conclusions are outlined in the final section.

## 2. Theories and Hypotheses

### 2.1. Literature Review and Institutional Background

Extant studies have discussed the strategic motivations for corporate philanthropy [14,17,20,22,23]. Corporate philanthropy is normally considered as one of effective non-market strategies in improving corporate reputation, corporate performance or shareholder wealth [17,24,25,26,27]. The strategic motivations are more likely to be connected with political legitimacy and resource acquisition in Chinese studies [14,20,28,29]. Gao and Hafsi, Li, Song and Wu pointed out that corporate philanthropy can ease the government’s burden in providing social welfare to society and it has been considered as an instrument to increase corporate political legitimacy and get support from the government [20,28]. Because of the unique institutional environment of China, most of important resources such as land, bank credit, subsidy, tax break are controlled by the government and restrict the development of firms [19]. In order to get political resources from the government, firms need to meet its expectations, especially the contribution of social welfare, so this may affect the strategy on corporate philanthropy. 

Furthermore, corporate environmental responsibility has been noticed in the studies of corporate philanthropy [2,9,12,14]. Several literature has studied the environmental concern as the hidden motivations for corporate philanthropy. On the one hand, firms donate on the environmental protection help to improve financial performance in a sustainable way via corporate reputation and stakeholder identity [2,4,9]. On the other hand, corporate philanthropic strategy may simply window-dressing to overshadow wrongdoing and cover the negative influence of their environmentally unfriendly behaviors [14]. Overall, limited literature suggests the relationship between corporate environmental responsibility and corporate philanthropy. In this study, we extend this line of literature and emphasize the role of resource and the government on this motivation in China. 

Previous literature has discussed the Chinese institutional effect on corporate strategies. In China, government plays an important role in corporate strategic decision. Government has the power reflecting on “... the rule of commerce; the structure of markets (through barriers to entry and changes in cost structures due to regulations, subsidies, and taxation); the offerings of goods and services that are permissible; and the sizes of markets based on government subsidies and purchases” [30]. Thus, government affects the firms and their market strategy and non-market strategy by implementing the functions on market regulation and resource distribution. Firstly, government is the first rule maker and enforcer. Firms need different levels of licenses to make business activities. Government conducts selective issuance of licenses through examination of corporate qualifications. Secondly, government controls most part of resources and distributes resources to firms. As the government needs to access information on firms in order to executive functions on regulation and resource distribution, some firms with good connections with government can obtain more benefits from the government, so firms make strategies to improve connections with government and establish political legitimacy. Several literature focusing on Chinese studies have proven this effective effect on the relationship between firms and the government [31,32]. Moreover, corruption is also a significant strategy on establishing and improving political connection [33]. Therefore, in Chinese study, government affects corporate strategies and institutional environment should be considered.

### 2.2. Corporate Environmental Responsibility and Corporate Philanthropy

During its transition period, China’s economy has developed rapidly and been transformed into the second largest in the world. However, the cost to the environment has been huge; it can be seen in air pollution, water pollution and soil pollution. The tension between environmental protection and economic development is deepening. In the face of worsening environmental problems and, in order to achieve sustainable development, the government continues to improve environmental regulatory policies to alleviate the current conflict between economic development and environmental protection.

In order to alleviate the serious issues caused by environmental problems, the government has taken a variety of measures with regard to environmental control. In order to comply with the various environmental regulations of local governments, firms invest many resources into energy-saving and emission-reduction. However, at present, with low levels of corporate environmental responsibility and relatively poor energy-saving and emission-reduction technologies, it is difficult to ensure that companies save energy and reduce emissions voluntarily. This means that firms are forced to invest many resources into energy-saving. This inevitably leads to a situation in which resources that are needed for the daily running of the firm go towards corporate environmental responsibility, which is not conducive to normal firm development. As rational economic agents, seeking for their own healthy and sustainable development, firms implement certain strategies to obtain social resources in order to make up for the loss caused by their non-spontaneous environmental behavior. Many previous studies have found that firms often resort to market development resources when they are facing with a shortage of resources or a deterioration of external environment, including obtaining some important resources through developing political connection, such as government subsidies, bank loans, tax incentives and so on [34,35,36,37].

In addition, the theory of resource dependence posits that firms are in an open system, that no firm can hold all the resources they need. In order to survive, firms need to draw resources from its surrounding environment [16]. In other words, organizations are interdependent; the informal links between organizations act as channels for persuasion and negotiation to stabilize these interdependent relationships. Organizations can implement strategies to manage their dependencies on each other, but their survival depends on the critical resources from the external environment. With the shortage capacity of environmental control, therefore, the government’s policies and regulations of environment force firms to take a certain strategic means to seek for the necessary resources for their own development while taking the passive environmental actions. 

As established in some of the previous literature on the strategic behavior of firms, philanthropy of contemporary enterprises is not driven by pure altruism and social responsibility [38]. To some extent, philanthropic strategy can help firms gain key resources. According to Zhang et al., the motivations for philanthropic strategy are mainly strategic and political [39]. Many pieces of research suggest that corporate philanthropy can help to enhance enterprises’ strategic positions and access to resources such as reputation capital, ultimately enhancing business performance [22,40]. On the other hand, firms may use corporate philanthropy to obtain the government’s goodwill and trust (i.e., “political capital”), to establish or maintain political relations and gain key strategic resources from the government [34,38].

Based on this logic, we believe that during China’s transitional period, in the context of government environmental regulation and the inability of enterprises to bear environmental responsibility, the environmental behaviors undertaken by enterprises can be non-spontaneous or passive environmental protection behavior. It is clear that these behaviors can easily lead to the loss of important resources, which is detrimental to the survival and development of the firm in question. Therefore, in order to ensure their survival and development, when fulfilling environmental protection obligations, firms use certain strategies to obtain resources to make up for those lost as a result of environmental problems. Corporate philanthropy is one major behavior strategy. Thus, we formulate Hypothesis 1:

**Hypothesis 1.**  There is a positive relationship between corporate eco-friendly events and corporate philanthropy.

### 2.3. Moderation Effect of Government Intervention

Many scholars believe that governments in emerging economies especially in China are more intervening in the market and corporate behavior than in developed countries [19,20]. The needs and visions of the local governments are endowed with their intervening activities and seeking for environmental protection is one of important political needs and social needs. In China under new situation, the contradiction between regional economic development and environmental protection is becoming more and more intense [41]. To a certain extent, the current emphasis on environmental protection limits the development of local traditional industries, a situation which creates a temporary conflict between environmental protection and regional economic development. Local governments need to take government intervention to invest extra resources in environmental protection and they are willing to encourage local firms to share this responsibility. Thus, firms participating in eco-friendly events are more likely to be perceived by local governments in areas where government intervention is stronger.

Moreover, local governments act as the provider of political resources [17,19,28]. The government in China controls critical resources and provides related resources to local firms through government intervention [20]. Corporate philanthropy, which helps local governments bear the social responsibility, has been proved to be an effective method to help firms obtain political legitimacy and critical resources from the government [17,20,28]. The premise of this process, however, is the perception of the government and the government favors the firms they are familiar with, so eco-friendly events that firms participate in are good stimulating signal. Therefore, based on this logic, firms participating in eco-friendly events are easier to be perceived by local government in a high-level government intervention area and this behavior will help firms obtain resources from local government more easily though corporate philanthropic strategy. Thus, we formulate Hypothesis 2:

**Hypothesis 2.**  Government intervention positively moderates the relationship between corporate eco-friendly events and corporate philanthropy.

### 2.4. Moderation Effect of Government Corruption

Government corruption, defined as the abuse of public power for private benefit, reflects the local institutional environment [42]. Government corruption could be easily seen in emerging economies, especially in China. During China’s transitional period, government departments have the right to allocate key resources in many fields, and these resources have an important influence on the survival and development of firms. Thus, obtaining these key resources always becomes an important part of corporate strategy, but the strategic design of access to key resources allocated by the government is affected by the local political environment. Based on the previous studies, in general, government corruption has two functions for firms, including ‘Protection Money’ and ‘Grease Money’. Therefore, the corruption behavior of double roles has a significant impact on corporate resource acquisition strategies and behaviors. That is, government corruption always help firms to use political connections with government officials to seek for political protection, further extortion and plunder, and achieve a resource allocation function [43,44].

In general, the more serious the government corruption in one region, the greater the likelihood that the environmental oversight system or official department will be manipulated by firms. In a more corrupt context, firms recognize that local government default corruption behavior is allowed. Using the strategy of bribery, the cost and risk of rent-seeking for firms is small and firms directly use local regulatory loopholes to bribe the relevant departments to alleviate the disadvantage of resources. Based on this logic, firms will adopt the strategy of bribery to local government, instead of corporate philanthropic strategy, thereby avoiding the need for corporate philanthropy to obtain resources to make up for the loss of resources brought about by environmental protection. Thus, we formulate Hypothesis 3:

**Hypothesis 3.**  Government corruption negatively moderates the relationship between corporate eco-friendly events and corporate philanthropy.

## 3. Data and Methodology

### 3.1. Sample and Data Collection

This study samples Chinese-based firms listed on Chinese Shanghai Stock Exchange and Shenzhen Stock Exchange from 2008 to 2013. We began with firms including 12,784 firm-year observations, of which 806 were discarded following selection procedures. Firstly, 794 firms receiving special treatment (ST and *ST) were removed because these firms have high-levels of risk, which may introduce pressures of rectification and delisting into the study and might improve their listing status via higher discretionary accrual [45,46]. Secondly, following the previous literature (e.g., Brammer and Millington), 12 firms were removed as the variables had missing values. At the final count, 11,978 firm-year observations from 2516 firms were used as a sample in this study [47].

Different sources of panel data were used in this study. Firstly, corporate financial data, including corporate eco-friendly events, were collected from the China Stock Market and Accounting Research (CSMAR) Database, a major database containing the information on Chinese stock markets and listed firms [17,28]. Secondly, corporate philanthropic data was hand-collected from corporate annual reports which were collected from the official websites of Stock Exchanges. Thirdly, provincial-level Gross Domestic Product (GDP) was collected from the China Statistical Yearbook [14]. Finally, government corruption data for each province was collected from the Procuratorial Yearbook of China.

### 3.2. Measurement of Variables

In this study, all variables are measured as follows.

#### 3.2.1. Corporate Philanthropy (Named Donation)

Corporate philanthropy is the main reflecting form of corporate social responsibility. In general, there are three kinds of measures for corporate philanthropy:

(1) Corporate philanthropic amount, measured as the corporate giving amount of one firm in a year, reflects the intensity on how firms participate in corporate philanthropy [17,48].
(2) Corporate philanthropic probability is measured as the dummy variables, coded as 1 if firms have displayed corporate philanthropic behavior in the year and otherwise coded as 0 [20,39]. The probability reflects the likelihood of adopting corporate philanthropic strategy.
(3) Corporate philanthropic ratio is measured as the amount of corporate philanthropy deflated by sales revenue or by total assets [20,49].
  In this study, we use the first method to test the hypotheses and robustness checks of our results.

#### 3.2.2. Corporate Eco-Friendly Events (Named Environment)

Corporate eco-friendly events, our independent variable reflecting corporate environmental responsibility in this study, is measured as the cumulative number of environmental protection events that firms engage in per year. These include events attempting to reduce carbon emissions, construct energy saving facilities, promote new environment-protecting technologies and other events on which firms expend resources. This method helps to measure the intensity of corporate environmental responsibility.

#### 3.2.3. Government Intervention (Named NERI)

Government intervention, one of the moderating variables in this study, is measured as the Marketization Index of Chinese Province from 2008 to 2014 by Wang et al. [50]. The Marketization Index represents the role of the government in business activities at the provincial level, and a higher marketization index means a lower level of intervention by local government within one region [51,52]. Thus, we use this index as the measure of government intervention in this study.

#### 3.2.4. Government Corruption (Named Corruption)

Government corruption is another moderating variable in this study. Government corruption is one of the main institutional environmental variable which is considered as the political strategy of Chinese firms. Government corruption is calculated as the total number of corruption cases brought against civil servants in one province and then given as the number of corruption cases per 10,000 civil servants [53].

#### 3.2.5. Control Variables

Following the previous literature, several control variables were chosen in this study:

(1) Firm size (named Firm size), measured as the logarithm of corporate total assets, has been proven to play an important role in corporate philanthropic behavior [47]. Larger firms have more resources to practice corporate philanthropy and tend to pay more attention to their reputations and shareholders’ attitudes [47,48,54].
(2) Firm age (named Firm age), measured as the number of years from a firm’s foundation until the end of 2013. Older firms have been proven to be likelier to accumulate more resources from different channels and to practice corporate philanthropy. This is made possible by their more stable organizational structures and greater social relevance [17,20].
(3) Leverage (named Leverage) measured as the percentage ratio of total debts to total assets [55].
(4) State ownership (named Stateshare), measured as the percentage of state share to the whole share. The value is equal to 0 if there is no state share in one firm [17,28].
(5) Corporate performance (named ROA), measured as the percentage value of Return to Assets (ROA). Previous studies have proved that firms with good financial performance are more likely to practice corporate philanthropy [17].
(6) Regional GDP (named GDP), measured as the logarithm of Gross Domestic Product (GDP) at the provincial level in China [56].
(7) Finally, we introduced Year dummy for each year from 2008 to 2013 into the regression.

### 3.3. Statistical Analysis

The panel data used in this study has hierarchical structures such that the variables government intervention, government corruption and regional GDP are at the provincial level, whereas other variables regarding corporate finance are at the firm level. Thus, the ordinary least square (OLS) regression is not suitable in this study because it would cause errors of constant variance and independence, so multilevel regression analysis is used instead [57]. In the operating process, we use “xtmixed” in STATA version 14.0 (StataCorp, College Station, TX, USA) in order to conduct mixed-effect multilevel linear regression. “xtmixed” is one specific command statement of the STATA software and is used to do multilevel analysis. Our statistical analyses are separated into different models, so that control variables are included into the regression first followed by the independent and moderating variables. As the variable regional GDP is at the provincial level, multilevel regression is used first. Moreover, OLS regression was used for robustness check in order to check whether the results are stable or not. If the results obtained from two different methods are the same, we can ensure that the results from the analysis are robust. Both Multilevel regression and OLS regression analysis are used by separate hierarchical regression analyses. Control variables are tested first before independent variable and moderating variables enter. The effect of each variable on dependent variable could be parceled out of the model [57]. Furthermore, considering the degree of multicollinearity, we calculated the variance inflation factors (VIFs). The results are distributed from 1.04 to 1.74 and the mean value is 1.32, significantly less than the critical value 10.00, so multicollinearity is not an important issue in this study [58].

## 4. Results

### 4.1. Descriptive Statistics

Table 1 reports the characteristics of the sample including max, min, mean, number of observations and percentage of total sample. Among the sample observations, 8034 ones (67.07% of the total) participate in corporate philanthropy and the largest logarithmic value of corporate philanthropy is 20.26 with a mean of 8.43. Regarding state ownership, 3542 observations (29.57% of the total) have state ownership and the largest percentage of state ownership is 0.92 with a mean of 0.09. 1763 observations (14.72) have eco-friendly events and the largest number of corporate eco-friendly events is 672 with a mean of 1.39. Government intervention, measured as the marketization index, ranged from the value −0.30 to 11.80 and the mean is 7.6. Government corruption ranged from the value 2.65 to 46.30 and the mean is 22.76.

Table 2 summarizes the descriptive statistics (means, and standard deviations) and Pearson correlation matrices for all variables in this study. The standard deviation of corporate philanthropy is 6.15, and the standard deviation of corporate eco-friendly events is 9.26. As would be expected, eco-friendly events are significantly correlated with corporate philanthropic giving. All variables are found to have a positive correlation with corporate philanthropy except firm age.

### 4.2. Regression Analyses and Results

In Table 3, Model 1 includes only the basic control variables and moderating variables. Firm size (*p* < 0.001), corporate performance (*p* < 0.001), government intervention (*p* < 0.001) and government corruption (*p* < 0.001) are positively associated with corporate philanthropy. Firm age (*p* < 0.05) and state ownership (*p* < 0.05) are negatively associated with corporate philanthropy. Model 2 tests the effect of corporate eco-friendly events (Hypothesis 1). Consistent with Hypothesis 1, eco-friendly events are positively associated with corporate philanthropy. In the model, the coefficient of eco-friendly events is positive and significant under the 10% level (*p* < 0.10), suggesting that eco-friendly events matter to and increase corporate philanthropy. The change of χ square in Model 2 is more significant than that in Model 1. Thus, there is evidence for Hypothesis 1.

Model 3 tests the moderating effect of government intervention. In order to weaken the adverse effects of multicollinearity and ensure the results were credible, the interactions were calculated as a standardized independent variable and a moderating variable. Hypothesis 2 predicts that government intervention weakens the positive relationship between eco-friendly events and corporate philanthropy. In the model, the interaction term of eco-friendly events and government intervention is significant (*p* < 0.05) and the coefficient of the term is negative, indicating that government intervention does weaken the positive association between eco-friendly events and corporate philanthropy. Furthermore, the χ square in Model 3 is more significant than that in Model 2 and the intra-class correlation (ICC) is about 0.4, which is the acceptable value of reliability for multilevel analysis. Thus, there is evidence for Hypothesis 2. In Model 4, we included the interaction term of eco-friendly events and government corruption. Hypothesis 3 predicts that government corruption weakens the positive relationship between eco-friendly events and corporate philanthropy. In the model, the interaction term is significant (*p* < 0.05) and the coefficient of the term is negative, indicating that government corruption weakens the positive association between eco-friendly events and corporate philanthropy. Considering χ square and the ICC value in Model 4, there is evidence for Hypothesis 3. Lastly, we included all interaction terms in Model 5 to test the stability of the results. The results of the coefficient and the p value are the same as the previous results in Models 3 and 4, indicating that these results have the characteristic of stability.

**Figure 1:**
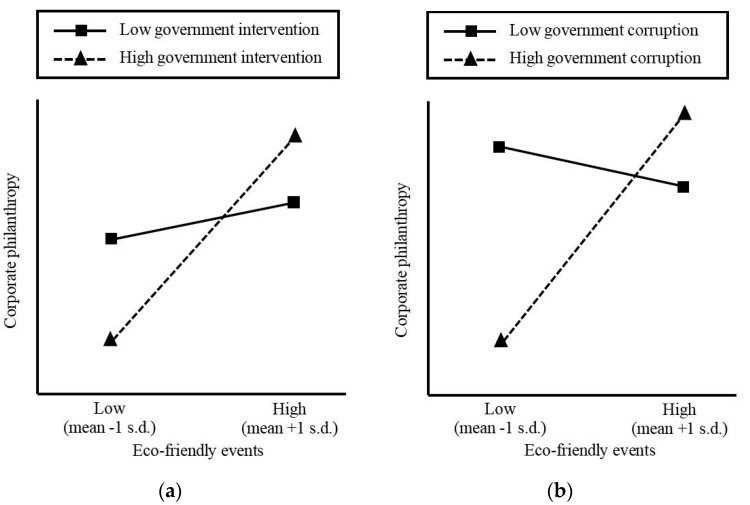
Graphic presentations of moderating effect of government intervention and government corruption in the regression. Specifically: (**a**) the moderating effect of government intervention; (**b**) the moderating effect of government corruption.

In order to demonstrate the moderating effects of government intervention and government corruption, we use Figure 1a,b to show the relationship between eco-friendly events and corporate philanthropy at the different levels of the moderating variables [17,41]. In Figure 1a, we can see the moderating effect of government intervention. As eco-friendly events increase by one standard deviation, corporate philanthropy also increases but this growing trend is more pronounced under conditions of low government intervention than it is under conditions of high government intervention, indicating that government intervention weakens the relationship between eco-friendly events and corporate philanthropy. Figure 1b shows the moderating effect of government corruption. As eco-friendly events increase by one standard deviation, corporate philanthropy increases under conditions of low government corruption but decreases under conditions of high government corruption, indicating that government corruption weakens the relationship between eco-friendly events and corporate philanthropy. Thus, the moderating effects shown in this study are both statistically and practically significant.

### 4.3. Robustness Checks

As the statistical analysis was tested by multilevel regression analysis, we use OLS linear regression to examine the results of the main effect and interaction terms by way of the robustness checks. The results of the five models are shown in Table 4. Control variables and moderating variables were entered into Model 1. Model 2 tested the main effect between eco-friendly events and corporate philanthropy; the results show that the coefficient of eco-friendly events is positive and significant (β = 0.01, *p* < 0.10), similar to the result of the multilevel analysis. Models 3 and 4 tested the moderating variables and government intervention is negatively significant (β = −0.30, *p* < 0.01). 

However, government corruption is negative but not significant in the confidence interval from 0 to 0.1. The result is less than 0.2 and the difference is not quite significant, meaning that this result can also be considered similar to the previous one due to the different calculation methods involved in the analyses. The values of adjust R square in five models equal 0.11, which reflects enough explanatory power of the whole regression in the topic of corporate philanthropy (e.g., 0.115 in the literature of Li and Zhang, 0.129 in the literature of Du) [19,29]. Thus, the checks confirm that the results are robust.

## 5. Discussion

The purpose of this study is to examine the relationship between corporate environmental responsibility and corporate philanthropy as well as the moderating effect of institutional environmental variables, including government intervention and government corruption. After the empirical test using Chinese listed firms in the period from 2008 to 2013, we find that corporate environmental responsibility significantly affects corporate philanthropy; specifically, corporate eco-friendly events enhance corporate philanthropy. This result supports our assumptions regarding environmental events held by firms and what motivates corporate philanthropic strategy. Based on the theory of resource dependence, firms that engage in eco-friendly events expend a larger quantity of resources for instance through investment in environmental protection facilities and capacity constraints [59]. Thus, firms that spend more resources on environmental responsibility are more motivated to engage in philanthropic activities in order to obtain the necessary resources controlled by the local government.

We also find that government intervention and government corruption negatively moderate the relationship between corporate eco-friendly events and corporate philanthropy, supporting our assumptions regarding the moderating effect of the Chinese institutional environment on corporate strategy. Government intervention is sensitive to the firms participating in eco-friendly events and firms can obtain more resources from the government by using corporate philanthropic strategy based on the sensitive connections between local government and firms. Government corruption, considered as direct mutually beneficial ties between firms and government, can help firms gain key resources from the government. Firms seek for more direct strategy of corruption rather than indirect corporate philanthropic strategies when there are conditions of high government corruption.

### 5.1. Contributions and Implications

This study contributes to the existing literature in several ways. Firstly, to the best of our knowledge and literature in hand, this study is one of the few to examine the relationship between corporate environmental responsibility and corporate philanthropy from an empirical perspective. More precisely, this study is the first to look at the direct relationship between corporate eco-friendly events (i.e., doing good) and corporate philanthropy. Previous literature (e.g., Du) has studied the positive association between corporate environmental misconduct and corporate philanthropy, where the motivation for corporate philanthropic strategy is to overshadow environmental wrongdoing [2,14]. In our study, we expand the focus of corporate environmental responsibility from only misconduct (or eco-harmful events) to include eco-friendly events and argue that firms that help the environment (i.e., by holding eco-friendly events) have a positive effect on the strategy of corporate philanthropy. Considering the fact that dependence on resources plays an important role in this relationship, the theory of resource dependence is addressed in the context of environmental responsibility studies. Therefore, this study broadens the study scope of the relationship between corporate environmental responsibility and corporate philanthropy.

Secondly, following some of the previous literature (e.g., Gao and Hafsi; Li, Song and Wu; Li and Zhang), we find that Chinese institutional environment plays an important role in corporate philanthropy and that it negatively moderates the positive association between corporate environmental responsibility and corporate philanthropy [20,28,29]. Institutional theory has previously noted the role of local government in shaping corporate strategy and behavior [20,60,61]. Previous literature has discussed the role of institutional variables such as political ties between corporate managers and the government, industry peers’ giving and industry-level discretion [20,28,57]. In this study, we expand on this and establish that government intervention and government are proven to have a moderating effect on the relationship between corporate environmental responsibility and corporate philanthropy.

Finally, regarding our research methods, few studies have previously brought the hierarchical structures found at the levels of firms and governmental institutions into corporate philanthropic studies. Focusing on the role of regional government, corporate strategies and behaviors such as environmental responsibility and philanthropic giving are one-to-one matched with their local institutional conditions in each region. Thus, it is necessary to investigate how institutional-level environment affects firm-level corporate strategies and behaviors, especially in the context of emerging markets [62]. Accordingly, using the context of China, our findings China can better reveal institutional effects on corporate responsibility.

### 5.2. Limitations and Future Study Directions

Our research has a few limitations which suggest directions for future research. Firstly, although we have clearly elaborated the motivations for firms that engage in eco-friendly events to participate in corporate philanthropic strategy from the theoretical perspectives of resource dependence and institutional environment, these strategies still rely on political motivations. Some scholars have made the point that, rather than political concerns, other motivations for corporate philanthropic strategy (e.g., firm reputation) may affect corporate choices on philanthropic strategy [63,64]. Future studies are needed to examine whether political concerns are indeed the main motivation for corporate philanthropic strategy.

Secondly, our study uses the number of corporate eco-friendly events per year as the measurement of the intensity of corporate environmental responsibility. This measurement can reflect the positive effects of corporate participation on environmental responsibility but not the amount of corporate resources used. Because we study this issue from the perspective of resource dependence, it does not well illustrate the changes in resource flow. Future studies may find a better method to measure the amount of resources that firms expend on environmental responsibility.

Thirdly, we have tested the positive relationship between corporate environmental responsibility and corporate philanthropy from a perspective of resource dependence, assuming that firms can gain key resources from the government by participating in philanthropic strategy. Previous research has proved that firms that engage in corporate philanthropic strategy can more easily gain resources from the government, but what kinds of resources and in what quantities are unclear [19,28]. An examination of these issues may provide deeper insights into how much political benefit is influenced by corporate philanthropy. Thus, procurement of resources from the government via the strategy of corporate philanthropy is an important question for future research to address.

## 6. Conclusions

By analyzing data from Chinese listed firms from 2008 to 2013, we have better demonstrated that firms participating in eco-friendly events are more likely to make corporate philanthropic strategy in order to obtain valuable resources from the government to make up for the loss of investment in environmental protection. The government acts as an important moderating system in which government intervention positively moderates the relationship and government corruption has a negative moderating effect. We hope this study can provide a better understanding of the hidden relationship between corporate eco-friendly events and corporate philanthropy. Future studies can bring new insights by figuring out and examining more substantial and more detailed influencing influential factors on the relationship between corporate eco-friendly events and corporate philanthropy.

## Figures and Tables

**Table 1:** Characteristics of the sample.

Characteristics	Max	Min	Mean	Number of Observations	% of Total N
Donation					
Have	1	1	1	8034	67.07
Not have	0	0	0	3944	32.93
Donation amount (log)	20.26	0	8.43	11,978	100
Firm size (log)	30.57	15.58	21.86	11,978	100
Firm age	33	1	13.05	11,978	100
Leverage (%)	1.27	0.01	0.45	11,978	100
Stateshare					
Have	1	1	1	3542	29.57
Not have	0	0	0	8436	70.43
Stateshare amount (%)	0.92	0	0.09	11,978	100
ROA (%)	0.94	−0.10	0.05	11,978	100
GDP (log)	11.04	5.98	9.95	11,978	100
Environment					
Have	1	1	1	1763	14.72
Not have	0	0	0	10,215	85.28
Environment amount	0	672	1.39	11,978	100
NERI	11.80	−0.30	7.60	11,978	100
Corruption	46.30	2.65	22.76	11,978	100

**Table 2:** Descriptive statistics and correlation matrix Dependent.

	Variables	Mean	SD	1	2	3	4	5	6	7	8	9	10
1	Donation	8.43	6.15	1									
2	Firm size	21.86	1.43	0.18	1								
3	Firm age	13.05	5.21	−0.02	0.15	1							
4	Leverage	0.45	0.22	0.08	0.52	0.28	1						
5	Stateshare	0.09	0.18	0.03	0.20	−0.07	0.12	1					
6	ROA	0.05	0.07	0.08	−0.11	−0.18	−0.40	−0.02	1				
7	GDP	9.95	0.76	−0.02	−0.04	−0.01	−0.12	−0.20	0.07	1			
8	Environment	1.39	9.26	0.05	0.18	0.02	0.05	0.01	0.01	0.01	1		
9	NERI	7.60	1.89	0.04	−0.02	−0.09	−0.07	−0.06	0.07	0.58	−0.01	1	
10	Corruption	22.76	6.64	0.01	−0.10	0.10	0.07	0.01	−0.08	−0.03	−0.03	−0.03	1

N = 11,978; correlations > |0.018| are significant at 0.05 level (2-tails).

**Table 3:** Multilevel regression on corporate philanthropy.

Variables	Model 1 β (SE)	Model 2 β (SE)	Model 3 β (SE)	Model 4 β (SE)	Model 5 β (SE)
Constant	−185.30 (207.13)	−170.39 (208.15)	−188.79 (207.38)	−172.04 (207.98)	−190.75 (207.17)
Firm size	0.91 *** (0.05)	0.89 *** (0.05)	0.88 *** (0.05)	0.88 *** (0.05)	0.87 *** (0.05)
Firm age	−0.02 * (0.01)	−0.02 ^+^ (0.01)	−0.02 ^+^ (0.01)	−0.02 ^+^ (0.01)	−0.02 ^+^ (0.01)
Leverage	0.50 (0.33)	0.52 (0.33)	0.54 (0.33)	0.54 (0.33)	0.55 ^+^ (0.33)
Stateshare	−0.68 * (0.32)	−0.67 * (0.32)	−0.67 * (0.32)	−0.67 * (0.32)	−0.66 * (0.32)
ROA	9.35 *** (0.82)	9.35 *** (0.82)	9.33 *** (0.82)	9.36 *** (0.82)	9.33 *** (0.82)
GDP	−0.23 (0.77)	−0.19 (0.77)	−0.25 (0.77)	−0.18 (0.77)	−0.25 (0.77)
NERI	0.75 *** (0.09)	0.76 *** (0.09)	0.75 *** (0.09)	0.76 *** (0.09)	0.75 *** (0.09)
Corruption	0.18 *** (0.04)	0.18 *** (0.04)	0.18 *** (0.04)	0.18 *** (0.04)	0.18 *** (0.04)
Year dummy	Include	Include	Include	Include	Include
Environment		0.01 ^+^ (0.01)	0.02 ** (0.01)	0.01 * (0.01)	0.02 ** (0.01)
Environment × NERI			−0.24 * (0.11)		−0.24 * (0.11)
Environment × Corruption				−0.21 * (0.10)	−0.21 * (0.10)
χ square	729.24 (10) ***	733.04 (11) ***	737.10 (12) ***	738.07 (12) ***	742.22 (13) ***
ICC at province level	0.39	0.39	0.39	0.39	0.39

N = 11,978; ^+^ *p* ≤ 0.10; * *p* ≤ 0.05; ** *p* ≤ 0.01; *** *p* ≤ 0.001 (2-tails).

**Table 4:** Robustness checks for OLS linear regression on corporate philanthropy.

Variables	Model 1 β (SE)	Model 2 β (SE)	Model 3 β (SE)	Model 4 β (SE)	Model 5 β (SE)
Constant	−8.48 *** (1.34)	−8.20 *** (1.35)	−7.78 *** (1.35)	−8.11 *** (1.35)	−7.68 *** (1.36)
Firm size	0.82 *** (0.05)	0.81 *** (0.05)	0.79 *** (0.05)	0.80 *** (0.05)	0.79 *** (0.05)
Firm age	−0.05 *** (0.01)	−0.05 *** (0.01)	−0.05 *** (0.01)	−0.05 *** (0.01)	−0.05 *** (0.01)
Leverage	1.00 ** (0.32)	1.02 ** (0.32)	1.04 *** (0.32)	1.03 *** (0.32)	1.04 *** (0.32)
Stateshare	−1.77 *** (0.32)	−1.76 *** (0.32)	−1.76 *** (0.32)	−1.76 *** (0.32)	−1.75 *** (0.32)
ROA	11.78 *** (0.81)	11.78 *** (0.81)	11.75 *** (0.81)	11.78 *** (0.81)	11.75 *** (0.81)
GDP	−0.10 (0.10)	−0.10 (0.10)	−0.11 (0.10)	−0.10 (0.10)	−0.11 (0.10)
NERI	−0.02 (0.04)	−0.02 (0.04)	−0.02 (0.04)	−0.02 (0.04)	−0.02 (0.04)
Corruption	0.01 (0.01)	0.01 (0.01)	0.01 (0.01)	0.01 (0.01)	0.01 (0.01)
Year dummy	Include	Include	Include	Include	Include
Environment		0.01 ^+^ (0.01)	0.02 ** (0.01)	0.01 ^+^ (0.01)	0.02 ** (0.01)
Environment × NERI			−0.30 ** (0.11)		−0.31 ** (0.11)
Environment × Corruption				−0.12 (0.10)	−0.12 (0.10)
F value	110.08 ***	102.46 ***	96.17 ***	95.74 ***	90.27 ***
R square	0.11	0.11	0.11	0.11	0.11
Adjust R square	0.11	0.11	0.11	0.11	0.11

N = 11,978; ^+^ *p* ≤ 0.10; * *p* ≤ 0.05; ** *p* ≤ 0.01; *** *p* ≤ 0.001 (2-tails).
